# Improving the productivity of malic acid by alleviating oxidative stress during *Aspergillus niger* fermentation

**DOI:** 10.1186/s13068-022-02250-7

**Published:** 2022-12-29

**Authors:** Na Wu, Mingyan Xing, Yaru Chen, Chi Zhang, Yingfeng Li, Ping Song, Qing Xu, Hao Liu, He Huang

**Affiliations:** 1 School of Food Science and Pharmaceutical Engineering, Nanjing Normal University, Nanjing, 210023 China; 2 College of Life Sciences, Nanjing Normal University, Nanjing, 210046 China; 3 Tianjin Engineering Research Center of Microbial Metabolism and Fermentation Process Control, Tianjin University of Science & Technology, Tianjin, 300457 China; 4 College of Biotechnology and Pharmaceutical Engineering, Nanjing Tech University, Nanjing, 211800 China

**Keywords:** *Aspergillus niger*, Elastin peptide, Oxidative stress tolerance, L-Malic acid, Metabolic engineering

## Abstract

**Background:**

As an attractive platform chemical, malic acid has been commonly used in the food, feed and pharmaceutical field. Microbial fermentation of biobased sources to produce malic acid has attracted great attention because it is sustainable and environment-friendly. However, most studies mainly focus on improving yield and ignore shortening fermentation time. A long fermentation period means high cost, and hinders the industrial applications of microbial fermentation. Stresses, especially oxidative stress generated during fermentation, inhibit microbial growth and production, and prolong fermentation period. Previous studies have shown that polypeptides could effectively relieve stresses, but the underlying mechanisms were poorly understood.

**Results:**

In this study, polypeptides (especially elastin peptide) addition improves the productivity of malic acid in *A. niger*, resulting in shortening of fermentation time from 120 to 108 h. Transcriptome and biochemical analyses demonstrated that both antioxidant enzyme-mediated oxidative stress defense system, such as superoxide dismutase (SOD), catalase (CAT) and glutathione peroxidase (GPX), and nonenzymatic antioxidant system, such as glutathione, were enhanced in the presence of elastin peptide, suggesting elastin peptide relieving oxidative stresses is involved in many pathways. In order to further investigate the relationship between oxidative stress defense and malic acid productivity, we overexpressed three enzymes (*Sod1*, *CAT*, *Tps1*) related to oxidation resistance in *A. niger*, respectively, and these resulting strains display varying degree of improvement in malic acid productivity. Especially, the strain overexpressing the *Sod1* gene achieved a malate titer of 91.85 ± 2.58 g/L in 96 h, corresponding to a productivity of 0.96 g/L/h, which performs better than elastin peptide addition.

**Conclusions:**

Our investigation provides an excellent reference for alleviating the stress of the fungal fermentation process and improving fermentation efficiency.

**Supplementary Information:**

The online version contains supplementary material available at 10.1186/s13068-022-02250-7.

## Introduction

Malic acid is widely utilized as a flavor enhancer and acidulant in the food industry, as a drug additive in pharmaceuticals and as metal cleaning paint in the field of the daily chemical industry [1, 2]. Malic acid is considered one of the 12 “building block” chemicals for the production of biodegradable polymers by the U.S. Department of Energy [3]. Annually, the application of malic acid in the global market has increased by approximately 4%, and the annual market is expected to be  > 200,000 tons [4]. Malic acid is mainly prepared by enzymatic approaches, in which fumaric acid and water are converted into malic acid by fumarate hydratase [5]. Fumaric acid was usually derived from petroleum-based products, which could result in potential safety hazards regarding the final product [6, 7]. Microbial fermentations to produce malic acid can employ various microorganisms and a large range of renewable substrates, which makes the process much more versatile and independent from fossil resources. Therefore, microbial fermentation to produce malic acid has attracted extensive attention in recent years [8, 9].

A large number of strategies have been explored to achieve the industrial application for malic acid fermentation. For instance, the malic acid production was 92.64 ± 1.54 g/L with the productivity of 0.48 g/L/h by *A. niger* PJR1 after 192 h [10]. *A. niger* PJR1 achieved the titer of 115.67 ± 3.5 g/L malic acid with the productivity of 0.53 g/L/h after 216 h in batch fermentation [11]. *A. niger* S575 obtained a titer 98.78 g/L malic acid in batch fermentation at 120 h, with a malic acid productivity of 0.82 g/L/h [12]. Those strategies mentioned above have high yields of malic acid, but are time-consuming. The long fermentation periods and high-cost are not applicable to industrialization process.

Previous studies have illustrated that there is intracellular stress in the process of microbial production, primarily induced by oxidative stress, product inhibition and nitrogen limitation, which finally deteriorating the fermentation performance of microorganisms and prolonging fermentation time [13]. For instance, the pentose utilization ability of yeast was decreased by oxidative stress [14–17]. The growth and production of *Escherichia coli* were inhibited by the acidic fermentative products or by-products [18]. In addition, nitrogen limitation stress exceeding a certain range resulted in decreasing the production of fumaric acid in *Rhizopus oryzae* [19]. Therefore, alleviating stress is vitally important to guarantee the productive robustness of the cells [20, 21].

In recent years, supplementation of peptides has been reported to improve the products accumulation and stresses alleviation in microorganism. Soy peptides as nitrogen sources could enhance *Saccharomyces cerevisiae* cell growth under low-temperature stress conditions [22]. Collagen peptide could significantly improve the stress resistance of *Saccharomyces cerevisiae* against product inhabitation to enhance bioethanol production [23]. Our previous studies have also demonstrated that peptides addition could help stress and accelerate efficiency in *Rhizopus oryzae* during organic acid synthesis [24]. However, previous research only focused on accessing its effects on products accumulation. In addition, the specific mechanisms of the improvement of products accumulation by exogenous addition of peptides remain elusive.

**Fig. 1:**
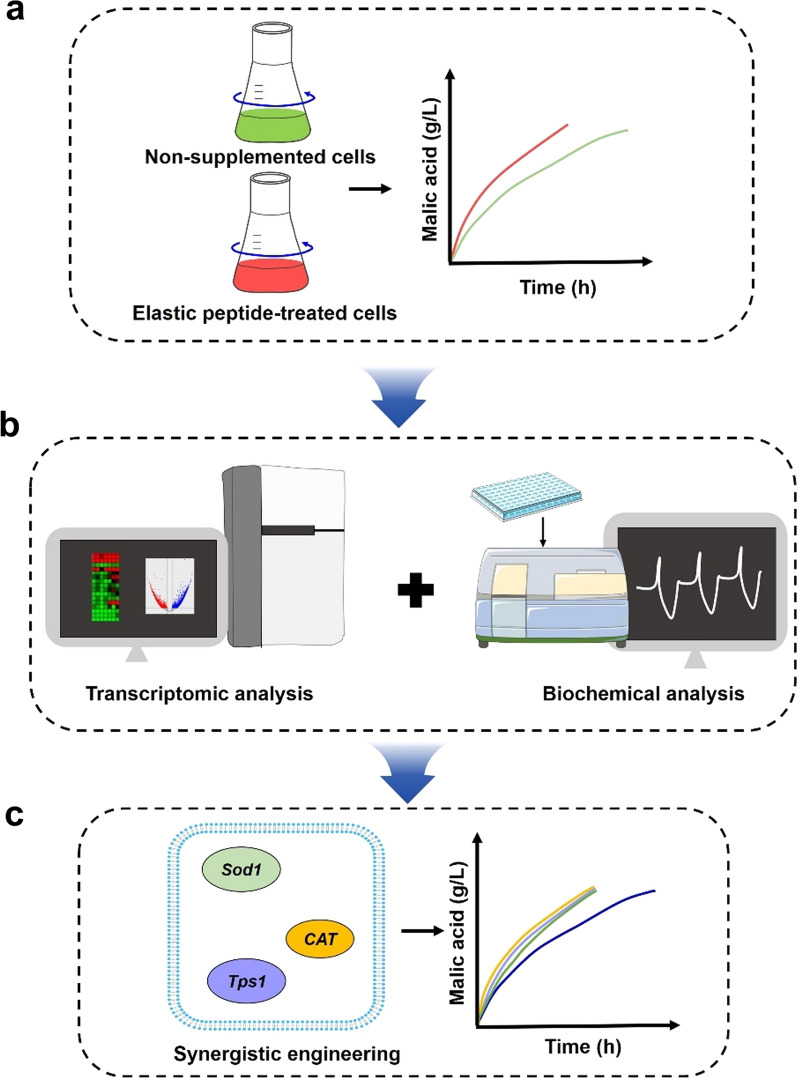
Schematic illustrating protein peptides relieving stress and enhancing the fluxes of glycolysis, r-tricarboxylic acid (TCA) and the glyoxylic acid bypass pathway in *A. niger* during malic acid bioproduction. **a** Fermentation assessment with peptides in *A. niger*; **b** transcriptomic analysis combined with biochemical analysis of elastin peptide supplementation in the fermentation period; **c** Overexpression of the gene for the stress-defense system and enhancement of the productivity of *A. niger*

Here, the mechanisms of stress tolerance induced by peptides in physiological and molecular levels were investigated, and a stress tolerance strain was developed to solve the problem of long fermentation time. We first confirmed that the addition of peptides could effectively increase the efficiency of malic acid fermentation, especially elastin peptide. Next, the possible mechanism of the elastin peptide in fermentation was demonstrated by transcriptome sequencing (RNA-seq) and biochemical assay analysis. The addition of elastin peptide upregulated the gene expression in the oxidative stress defense system and upregulated the gene expression in the malic acid synthesis pathway (Fig. 1b). Finally, based on the above analysis, we overexpressed the genes (*Sod1*, *CAT*, *Tps1*) related to the stress defense system of *A. niger*, and the fermentation results showed that recombinant strains could not only maintain the same yields, but also effectively shorten the fermentation duration from 120 to 96 h (Fig. 1c). Therefore, the productivity of malic acid could be enhanced by improving the stress tolerance of *A. niger*.

## Materials and methods

### Strains, medium and cloning

*Aspergillus niger* RG0095 (CCTCC M20221573) was preserved in the China Centre for Type Culture Collection (CCTCC), and was used in the present study as the starting strain. The strain was preserved in 20% (v/v) glycerol in a − 80 °C freezer. As in previous studies [25], *A. niger* RG0095 was cultured on potato-dextrose agar (PDA) at 28 ℃ for 5 days. Then, the spores of *A. niger* RG0095 (1 × 10^8^ spores/mL) were washed and transferred to 50 mL of fermentation medium (100 g/L glucose, 0.15 g of K_2_HPO_4_, 0.15 g of KH_2_PO_4_, 0.1 g of CaCl_2_·2H_2_O, 0.1 g of MgSO_4_·7H_2_O, 0.005 g of NaCl, 6 g of Bacto peptone, 0.005 g of FeSO_4_·7H_2_O and 80 g of CaCO_3_). Then we cultured them at 28 ℃ and 200 rpm for 120 h. The four commercially available polypeptides derived from elastin, silk, collagen, and keratin used in this study were purchased from Qingdao Youshunfa (99.8%, Qingdao Youshunfa Biotechnology Co., Ltd, China).

The *A. niger* RG0095 was utilized as the initial host [12]. The genes *Sod1*, *CAT* and *Tps1* were amplified from the genome of *A. niger* RG0095 and cloned by the primers showed in Table 1. The plasmid pEASY-Cre-LoxP-Pgpd containing the glyceraldehyde-3-phosphate dehydrogenase promoter (PgpdA) and loxP-hph-loxP cassette was used for gene integration and overexpression (Table 2). Plasmid pEASY-Cre-LoxP-Pgpd-*Sod1* was derived from pEASY-Cre-LoxP-Pgpd by overexpressing PgpdA with *Sod1*. Similarly, *CAT* and *Tps1* were individually used to obtain plasmids pEASY-Cre-LoxP-Pgpd-*CAT* and pEASY-Cre-LoxP-Pgpd-*Tps1* (Table 2). All restriction enzymes and DNA polymerase were bought from Takara (Shiga, Japan). The ClonExpressR II One Step Cloning Kit and T4 DNA ligase were bought from Vazyme (Nanjing, China) and Yeasen (Shanghai, China), respectively. DH5α was utilized as the overexpression host for plasmid construction and was cultured in Luria–Bertani (LB) broth added with 100 μg/mL ampicillin at 37 °C, 220 rpm for 12 h. The strains used in this study are listed in Table 3.

**Table 1:** Primers used in this study for plasmid construction

Primer	Sequence
*CAT*-F	ACCCTCACTAAAGGGCTATTTGCTACTCTCCCTCTT
*CAT*-R	AGACACATCTAAACAATGGATCAACGCTACTATACC
*Sod1*-F	ACCCTCACTAAAGGGTTAGGCGGAGAAGCGCTTCTC
*Sod1*-R	AGACACATCTAAACAATGGCTGCTTCCCTCGTCCGC
*Tps1*-F	ACCCTCACTAAAGGGTCACTGTGCCACCTGCTCCTG
*Tps1*-R	AGACACATCTAAACAATGCCTTCCCTCGAAAACCCC

**Table 2:** Plasmids used in this study

Plasmid	Description	Source
pEASY-Cre-LoxP-Pgpd	loxP-hph-loxP, gpdA promoter, trpC terminator, hygr, AMPr	This study
pEASY-Cre-LoxP-Pgpd-*Sod1*	loxP-hph-loxP, gpdA promoter, trpC terminator, hygr, AMPr, *Sod1* gene	This study
pEASY-Cre-LoxP-Pgpd-*CAT*	loxP-hph-loxP, gpdA promoter, trpC terminator, hygr, AMPr, *CAT* gene	This study
pEASY-Cre-LoxP-Pgpd-*Tps1*	loxP-hph-loxP, gpdA promoter, trpC terminator, hygr, AMPr, *Tps1* gene	This study

**Table 3:** Strains used in this study

Strain or plasmid	Genotype	Source
RG0095	Δ*oahA, pyc, mdhC, gpd::C4T318*	This study
RG0580	Δ*oahA, pyc, mdhC, gpd::C4T318, gpd::Sod1, hph*	This study
RG0581	Δ*oahA, pyc, mdhC, gpd::C4T318, gpd::CAT, hph*	This study
RG0582	Δ*oahA, pyc, mdhC, gpd::C4T318, gpd::Tps1, hph*	This study

All plasmids we constructed as vectors were able to randomly integrate into the genome of *A. niger* RG0095. The detail methods about the protoplast preparation and transformation and isolation of transformants were referred to the published article [26].

### Measurement of malic acid and glucose

As in previous studies [27], in brief, 15 parallel shake flasks were set for each group during the fermentation process. We take 3 shake flasks from the shaker when taking samples every day. Then 2 ml of fermentation samples were transferred into a 10-mL tube each time, and the same volume of 2 M hydrochloric acid (HCl) was used to dissolve excess CaCO_3_. Then, the mixture was added to 2-mL tubes and centrifuged at 11000 rcf for 10 min. The supernatant was diluted in ultrapure water and filtered through a 0.22-μm filter membrane for high-performance liquid chromatography (HPLC) analysis, quantified by HPLC using an Aminex HPX-87H column (Bio-Rad, Hercules, USA) and an ultraviolet (UV) detector at 210 nm. The mobile phase consisted of 5 mM H_2_SO_4_ at a flow rate of 0.6 mL/min. In addition, the injection volume of HPLC was set as 20 μL and the column temperature was set as 65 ℃.

The glucose concentration was measured by a SBA-40C dual channel biosensor analyzer purchased from the Jinan Yanhe Biotechnology Company (Jinan, Shandong, China).

### RNA sequencing and data analysis

*A. niger* RG0095 was cultured in fermentation medium with 2 g/L elastin polypeptide and nonsupplemented at 28 ℃ and 200 rpm. After 72 h of cultivation, mycelia from the shake flasks were harvested for RNA extraction by TRIzol reagent (Invitrogen, Carlsbad, CA, USA). DNase I (Takara, Beijing, China) was used to remove genomic cDNA, and added to the extracted total RNA at 37 °C for 0.5 h. The TruSeq™ RNA Sample Prep Kit (Illumina, San Diego, CA, USA) was utilized to construct the cDNA library, and the RNA-seq libraries were constructed by an Illumina NovaSeq6000 platform from Major Bio Co., Ltd. (Shanghai, China). In order to obtain the differentially expressed genes (DEGs), the fragments per kilobases per million reads (FPKM) coupled with reads per kilobases per million reads (RPKM) were used in this study, then the p value was corrected by false discovery rate (FDR) and multi hypothesis testing. The differentially expressed genes were determined by |log_2_ (fold change) |≥ 1 and a false discovery rate (FDR) value  ≤ 0.05. Three biological replicates were conducted in each experiment.

### Enzyme assays

Mycelia from the flasks were collected after incubation for 72 h. The activities of the assays were tested by the test kits (Beijing Solarbio Science & Technology Co., Ltd., Beijing, China). Biochemical analysis of the stress defense system, such as H_2_O_2_ content, relative reactive oxygen species (ROS) level, superoxide dismutase (*SOD*) activity, catalase (*CAT*) activity, glutathione reductase (*GR*) activity, glutathione (GSH) content, glutathione S-transferase (*GST*) activity, glutathione disulfide (GSSG) content, total antioxidant capacity (T-AOC), trehalase (THL) activity, trehalose synthase (*TS*) activity, and the activity of the essential enzymes (phosphofructokinase (*Pfk*), hexokinase (*HK*), pyruvate kinase (*PK*), pyruvate carboxylase (*Pyc*) and malate synthase (*MS*)) in glycolysis, r-TCA and the glyoxylic acid bypass pathway were detected by test kits.

### RNA extraction and transcription analysis

Mycelia were collected from the fermentation broths after 72 h of cultivation for RNA isolation. Total RNA was extracted by the E.A.N.A. Fungal RNA Kit (Omega Biotek, Inc., Norcross, GA, USA) according to the manufacturer’s protocol. The RNA samples were utilized to synthesize cDNA with the PrimeScript RT Reagent Kit Perfect Real Time (Takara, Japan). The quantitative real-time reverse-transcription PCR (qRT-PCR) analysis was performed by the Applied Biosystems StepOneTM Real-Time polymerase chain reaction (PCR) System Thermal Cycling Block as described previously [26]. The calculated threshold cycle (Ct) for each gene amplification was normalized to the reference gene beta-actin. Finally, changes in gene expression levels between the EP-treated group and the group without supplementation were calculated by the arithmetic Formula 2^−ΔΔCt^. The primers used for qRT-PCR are shown in Table 4. We selected the actin protein-encoding gene as the reference gene. Each experiment was conducted with three biological replicates.

**Table 4:** The primers used for qRT-PCR in this study

Primer	Sequence
*CAT* F	TTGCAGAAGGCTGCCCCTACGC
*CAT* R	TCCAGATTTCCCTCATCCGTAT
*Sod1* F	TGACCTGTCCTACGACTATGGC
*Sod1* R	GGAGCAAGGTTCTCCCAGAAGA
*Sod2* F	TCAAGGCTGTCGCTGTTATCCG
*Sod2* R	TCACCGACGTGACGCTCGTCGT
*Tps1* F	TCAGAATGAGGCTCGCTTGCTC
*Tps1* R	GGCGTTATACTCCTGCTTCAA
*Pfk* F	AAGCTCCCGTGCAACCGCCCAA
*Pfk* R	CGGCGTCACAGATGCGAGTGAG
*Pyc* F	TCGATGACACCGAGTTCATCGAT
*Pyc* R	TCGTCGGCCTTCTGACGGTGCAT
*MS* F	ATGGTGCAAGTCGACACCCAAC
*MS* R	CGCTGGAATCCTCGAAATCAGC
ACTIN-F	CGGTAGGACGATCACATC
ACTIN-R	ACGATCAGCTTTCGGATGT

## Results

### Fermentation kinetics of *A. niger*

**Fig. 2:**
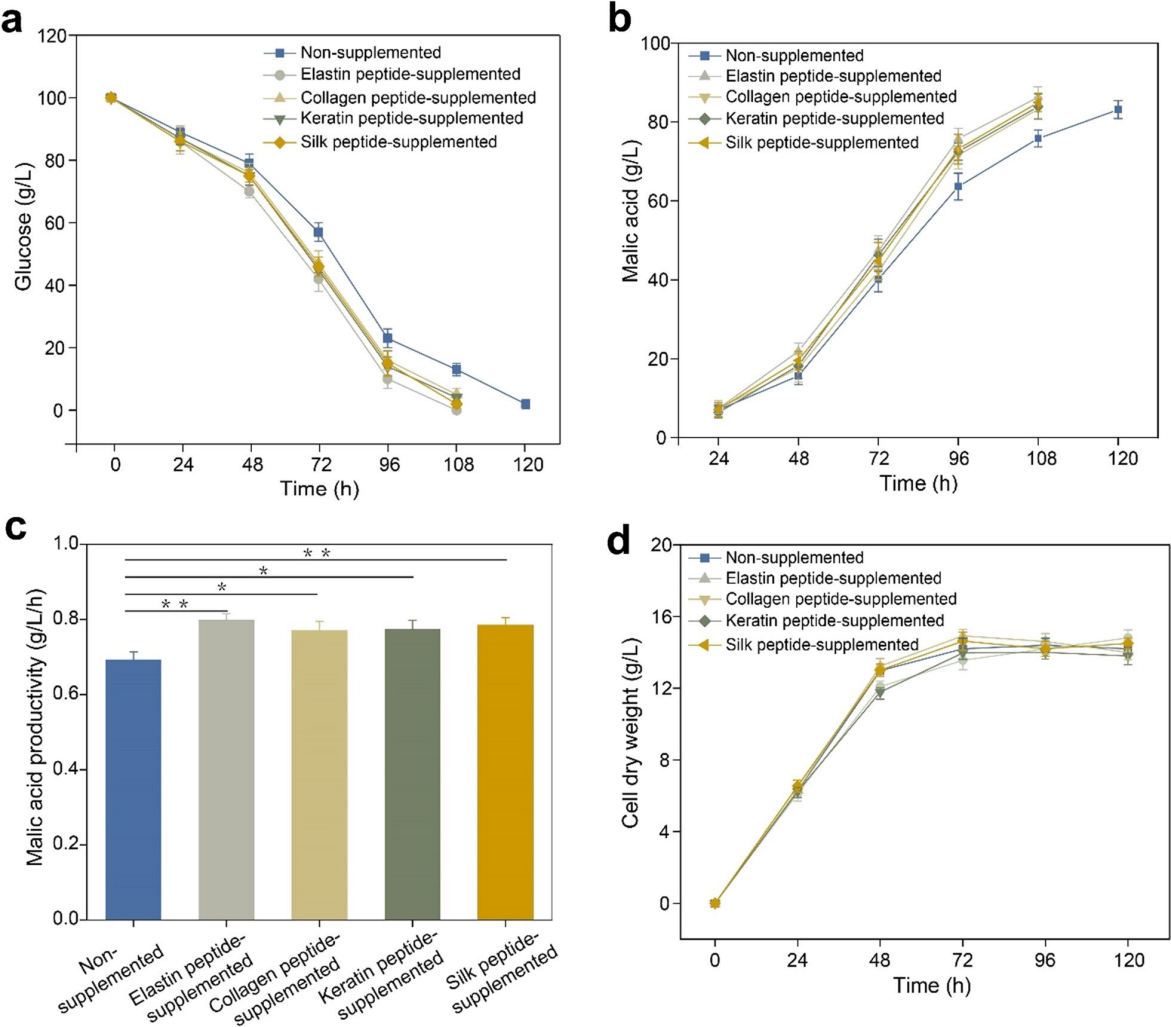
Fermentation kinetics of different peptides supplemented in *A. niger* RG0095 fermentation culture medium. **a** glucose consumption; **b** malic acid production; **c** malic acid productivity; **d** cell dry weight. Data represent the mean ± standard deviation (SD) of three independent replicates. Statistical significance was determined by Student’s *t*-test (*n* = 3). *p < 0.05, **p < 0.01

We first chose four commercially available peptides, collagen, silk, elastin, and keratin to evaluate their respective fermentation kinetics against *A. niger* RG0095. Then, we detected the glucose consumption of all the groups with 2 g/L peptides, and the results showed that the glucose consumption of all the groups with collagen, silk, elastin and keratin peptide was faster than the glucose consumption of the group without peptides (Fig. 2a). Obviously, the supplemented peptide group almost exhausted all glucose in 108 h, while the glucose in the nonsupplemented group was approximately 13 g/L (Fig. 2a). Therefore, we preliminarily believed that several peptides could promote the utilization of glucose in *A. niger* and effectively shorten the fermentation time.

Next, we tested the malic acid production of the peptide-supplemented groups and nonsupplemented group. The results demonstrated that the titer of malic acid in the peptide-supplemented groups was similar at 108 h (Fig. 2b), and it was also similar to the titer of malic acid in the nonsupplemented group at 120 h. Thus, the productivity of the peptide-supplemented groups was higher than the productivity of the nonsupplemented group, especially the productivity of the elastin peptide group (0.80 ± 0.02 g/L/h), which was nearly 15.9% (0.69 ± 0.03 g/L/h) higher than the productivity of the nonsupplemented group (Fig. 2c). In short, the addition of peptides during malic acid fermentation by *A. niger* accelerated the consumption of substrate (glucose) and shortened the fermentation time.

The cell dry weight (CDW) in all groups was similar and reached a stable stage after 72 h during fermentation. The CDW in elastin peptide-added group was 13.56 ± 0.52 g/L at 72 h, while the nonsupplemented group was 14.2 ± 0.59 g/L (Fig. 2d). Therefore, we inferred that the significant increase of L-malic acid productivity of *A. niger* was not due to the increase of biomass during fermentation, but might be due to the improvement of microbial metabolism by peptide addition.

Then, we chose elastin peptide with relatively good effects as the research object, and the optimum concentration of elastin peptide was determined in Additional file 1: Figure S1. The glucose uptake increased with the concentrations of peptide-supplemented during fermentation, especially between 1 and 2 g/L. However, the increase in glucose uptake was not obvious when the added concentration exceeded 3–4 g/L (Additional file 1: Figure S1a). We also found that the production of malic acid in the different concentrations of peptide-supplemented groups and the nonsupplemented group was almost the same (Additional file 1: Figure S1b). Considering the cost factor, 2 g/L peptides was chosen as the optimal concentration in the subsequent experiments.

### Transcriptome analysis

**Fig. 3:**
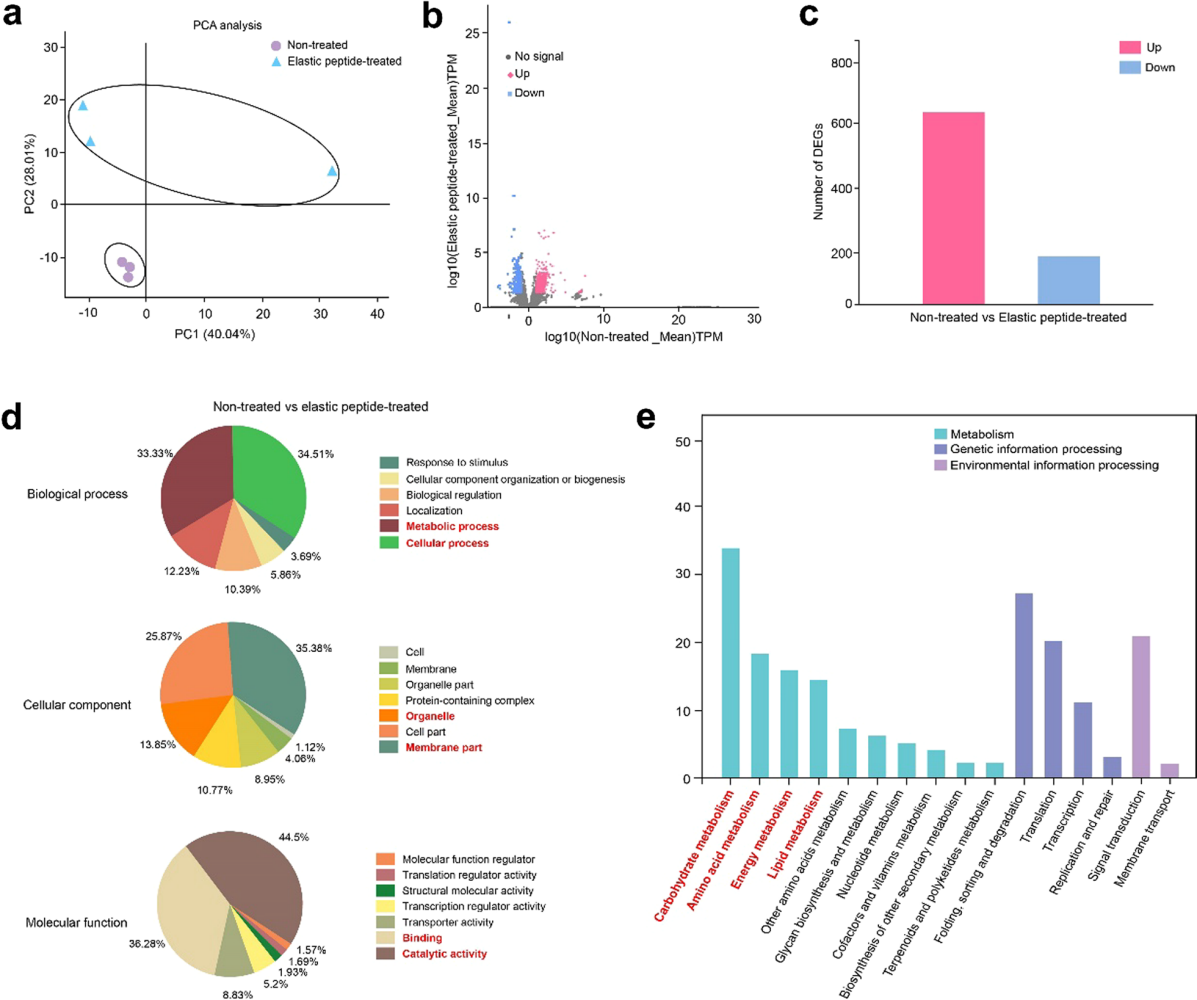
Transcriptomic profiling and annotation of differentially expressed genes between the fermentation medium with elastin peptide-supplemented group and the nonsupplemented group. **a** Score plots of the transcriptome of the elastin peptide-supplemented group compared to the nonsupplemented group; **b** volcano plots of the transcriptome of the elastin peptide-supplemented group compared to the nonsupplemented group; **c** up- and downregulated DEGs in the elastin peptide-supplemented group compared to the nonsupplemented group; **d** GO annotation of DEGs in the elastin peptide-supplemented group compared to the nonsupplemented group; **e** KEGG annotation of the DEGs in the elastin peptide-supplemented group compared to the nonsupplemented group

To further investigate the mechanism of elastin peptide in *A. niger* RG0095, transcriptome sequencing (RNA-seq) analysis was carried out for the elastin peptide-supplemented and nonsupplemented groups. We found that the genes from the group with elastin peptide and the nonsupplemented group were clustered into two distinct groups by principal component analysis (PCA), demonstrating that the transcriptional state of *A. niger* RG0095 was markedly affected by elastin peptide (Fig. 3a). According to the volcano plots, we found that the expression levels of large amounts of genes were down- or up-regulated in the elastin peptide-treated cells against the nonsupplemented cells (Fig. 3b). The expression levels of 813 genes were obviously changed (p-adjusted (FDR) < 0.05, |log_2_ (fold change) |> 1): 640 genes were upregulated, and 173 genes were downregulated (Fig. 3c, Additional file 2: Table S1). Gene ontology (GO) annotation analysis demonstrated that the 813 DEGs were classified into 20 functional groups, including biological process (6), cellular component (7) and molecular function (7) (Fig. 3d). In the cellular component category of GO annotation, the DEGs related to membranes and organelles accounted for an important position (Fig. 3d). Then, we found that large quantities of DEGs were related to the categories of mitochondria, endoplasmic reticulum, ribosomes, plasma membranes and vacuoles in elastin peptide (Additional file 1: Figure S2). The mitochondrion, as a power station, was a crucial site for oxidative phosphorylation and electron transport. Previous studies have shown that vacuole function is related to the stress protective function of a long series of proline in *Saccharomyces cerevisiae* [28]. Elastin peptide is composed of repeat sequences (valine-proline-glycine-valine-glycine), and proline is rich in elastin peptide [29, 30]. Therefore, the results implied that the vacuole and mitochondria might play a greater role in enhancing the stress tolerance supplied by elastin peptide. Further analysis revealed that the most altered genes were those related to catalytic activity, binding, cellular process and metabolic process (Fig. 3d). Therefore, GO annotation data also suggested that a large number of catalytic activities, in which multiple complex pathways were activated, and was changed by elastin peptide.

**Fig. 4:**
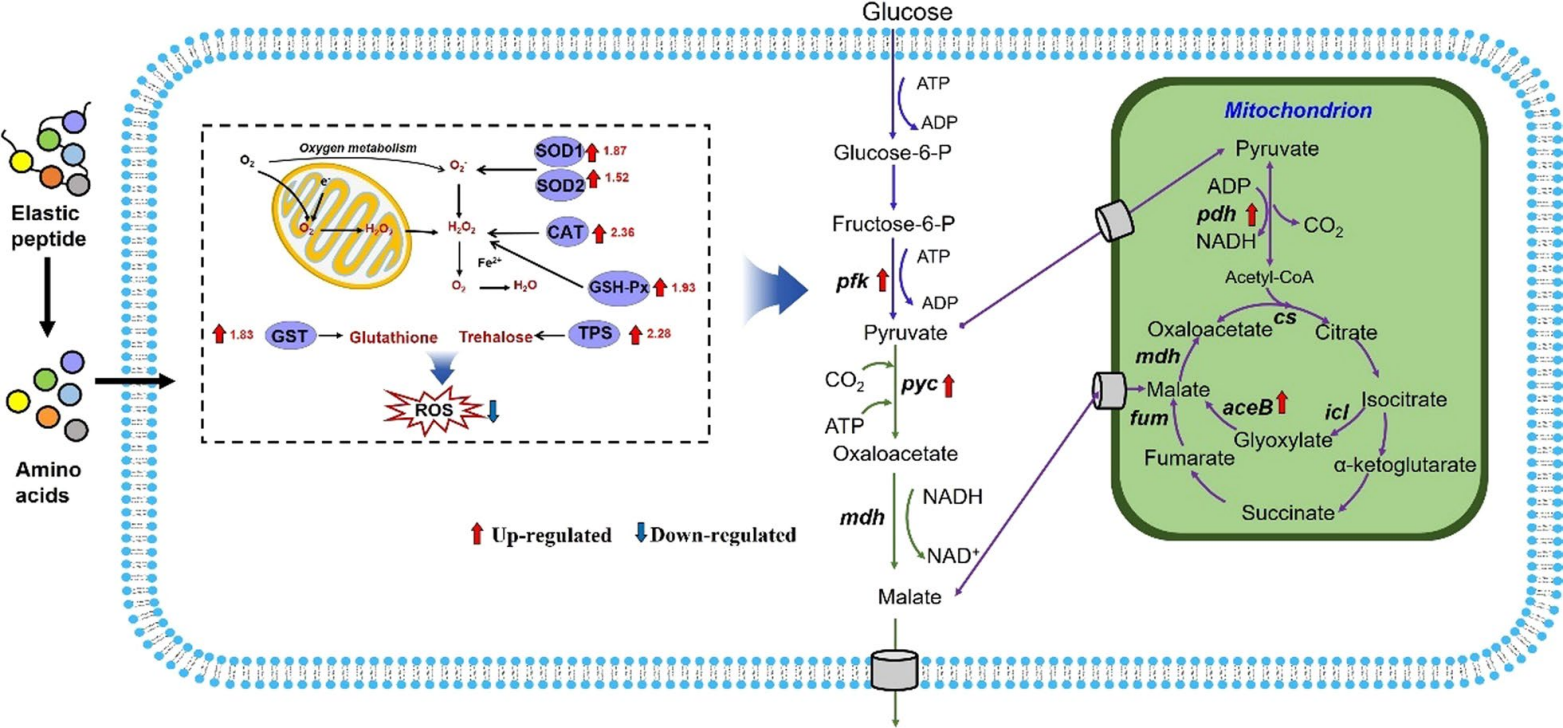
Proposed cell signal transduction pathway regulating malic acid production in *A. niger* during elastin peptide supplementation

We used Kyoto Encyclopedia of Genes and Genomes (KEGG) database to annotate and classify the expression profile of genes related to different metabolic pathways in the elastin peptide-supplemented and nonsupplemented groups. The results showed that numerous DEGs related to carbohydrate, amino acid, energy, and lipid metabolism (Fig. 3e). Previous studies have demonstrated that the lipid metabolism of yeast plays an important role in membrane structure and stress tolerance [31]. Consequently, combined with previous studies about the related mechanisms of peptides on fungi, we conjectured a framework of possible pathways consisting of two parts: a resistance to stress pathway and a malic acid synthesis pathway (Fig. 4).

Next, we investigated the expression profiles of genes related to the *A. niger* RG0095 stress defense system. From the transcriptome analysis, the genes encoding superoxide dismutase (*SOD*), such as the TRINITY_DN5829 gene encoding *Sod1* and the TRINITY_DN834 gene encoding sod2, were evidently upregulated 1.87- and 1.52-fold in the elastin peptide-supplemented group, respectively (Fig. 4, Additional file 2: Table S1). In addition, the expression levels of the TRINITY_DN5440 (encoding catalase) and TRINITY_DN6867 (encoding GSH-Px) genes were upregulated 2.36- and 1.93-fold compared to the expression levels of the genes in the nonsupplemented groups, respectively (Fig. 4, Additional file 2: Table S1). Therefore, the results demonstrated that elastin peptide might promote the synthesis of enzymes (e.g., *SOD*, catalase and GSH-Px) related to stress resistance, which could significantly enhance the stress resistance of *A. niger* against adverse environments and reduce cell damage.

Our transcriptome results showed that the expression level of glutathione-S-transferase (encoded by TRINITY_DN1421) in the elastin peptide-treated cells was upregulated by 1.83-fold compared with the glutathione-S-transferase in the nonsupplemented cells (Fig. 4, Additional file 2: Table S1). We found that the expression level of *Tps1* (encoded by TRINITY_DN4179) was increased by 2.28-fold after elastin peptide addition (Fig. 4, Additional file 2: Table S1). Therefore, we can infer that elastin peptide promotes the synthesis of cellular protectants (e.g., glutathione and trehalose) to enhance the stress resistance of cells to adverse environments.

Previous studies have demonstrated that the 6-phosphofructokinase encoded by the *pfk* gene is a potential limiting step for L-malate synthesis [26]. In our transcriptomic analysis, the expression level of *pfk* (encoded by TRINITY_DN920) was increased by 1.95-fold with elastin peptide addition (Fig. 4, Additional file 2: Table S1). Then, we detected gene changes involved in the reductive tricarboxylic acid (rTCA) pathway. Pyruvate carboxylase (*Pyc*) is one of the most critical enzymes for the synthesis of malic acid in the rTCA cycle. According to the transcriptomic results, the expression level of Pyc (encoded by TRINITY_DN2658) was upregulated by 1.32-fold in the elastin peptide-supplemented group (Fig. 4, Additional file 2: Table S1). There are two key enzymes in the glyoxylic acid bypass pathway for biosynthesizing malic acid: isocitrate lyase (*ICL*) and malate synthase (*MS*/*aceB*). Isocitric acid from the TCA cycle decomposes to glyoxylic acid under the action of ICL and then condenses with acetyl-CoA under the action of MS to form malic acid [32]. Our transcriptome analysis was in accordance with these reports, and the expression level of MS (encoded by TRINITY_DN9657) was upregulated by 6.97-fold in the elastin peptide-added cells (Fig. 4, Additional file 2: Table S1). Therefore, our transcriptome results fully demonstrated that the expression levels of these enzymes were significantly upregulated by addition of elastin peptide, which may accelerate glycolysis and the r-TCA and glyoxylic acid bypass pathways.

### Validation of RNA-Seq by qRT-PCR

**Fig. 5:**
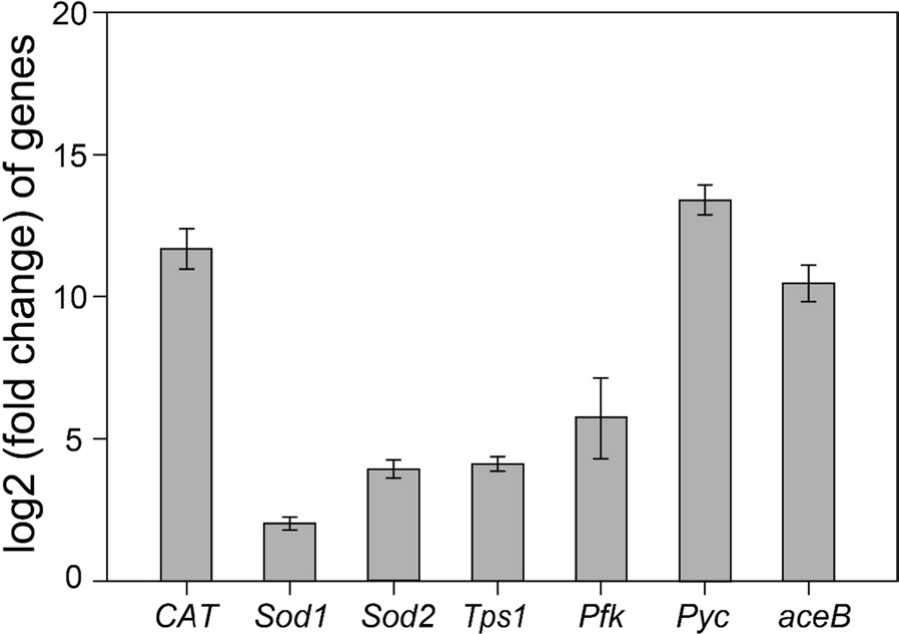
Validation of RNA-seq data using qRT-PCR analysis of the selected key genes. Error bars represent three technical replicates

The transcription profiles were confirmed by qRT-PCR experiment of the key genes involved in metabolism (Fig. 5) in nonsupplemented cells versus elastin peptide-treated cells. The results showed that the qRT-PCR data were in accordance with the results of transcriptomic profiles, which demonstrated that the RNA-seq results were convincing. It was worth noting that the expression profiles of genes related to the stress defense system (*Sod1*, *Sod2* and *CAT*) and the main pathways for malic acid synthesis (*pfk*, *pyc* and *aceB*) were upregulated in the elastin peptide-supplemented cells (Fig. 5). The results demonstrated a visible positive coherence between RNA-Seq transcriptomics and qRT-PCR results, showing a good quality of RNA-Seq results.

### Biochemical analysis

**Fig. 6:**
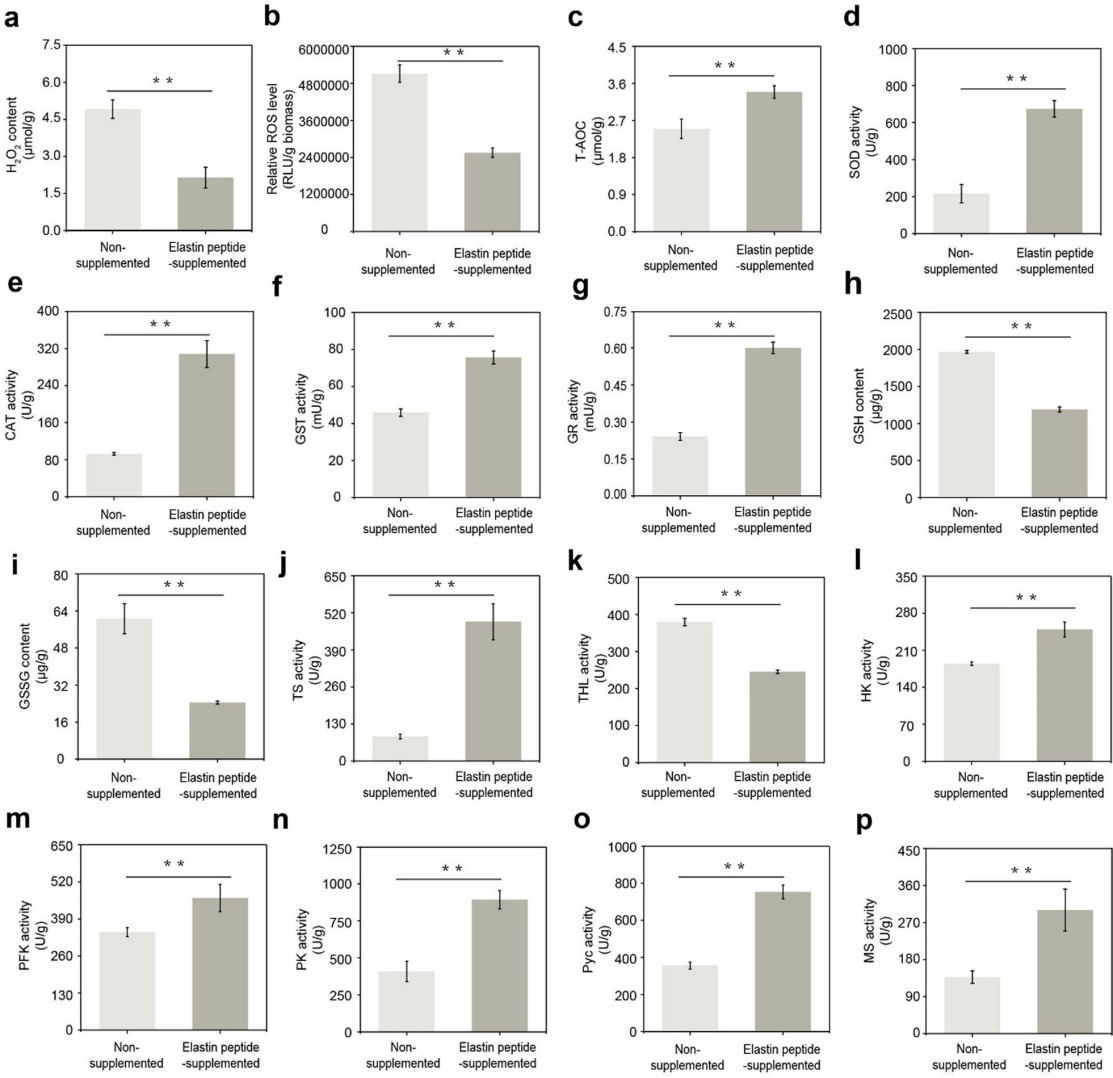
Biochemical analysis of the stress defense system in *A. niger*. **a** H_2_O_2_ assay; **b** ROS assay; **c** SOD assay; **d** CAT assay; **e** GST assay; **f** GR assay; **g** GSSH assay; **h** GSSG assay; **i** TS assay; **j** THL assay; **k** TOC assay; **l** HK assay; **m** Pfk assay; **n** PK assay; **o** Pyc assay; **p** MS assay. Statistical significance was determined by Student’s *t*-test (*n* = 3). **p < 0.01

Then, we further performed biochemical analyses to verify the transcriptome results. To test the influences of elastin peptide on the oxidative defense systems of *A. niger*, the dosages of H_2_O_2_ and ROS were determined. Compared with the nonsupplemented cells, we found that the H_2_O_2_ concentration was decreased in elastin peptide-added cells at 72 h (Fig. 6a). The H_2_O_2_ concentration and the relative ROS level in the elastin peptide-added group were significantly reduced by 56.5 and 50.2% at 72 h, respectively (Fig. 6a, b). These results indicated that the oxidative stress in *A. niger* was decreased by the elastin peptide to some extent. Total antioxidant capacity (T-AOC) refers to the total resistance of cells to environmental stress. Therefore, T-AOC levels were detected during fermentation. As shown in Fig. 6c, the T-AOC values of the cultures grown with supplemented elastin peptide reached 3.39 and were 35.6% higher than the T-AOC values of the cultures of the nonsupplemented group.

To further study the regulatory mechanisms mediating the observed effects of elastin peptide on stress resistance enzymes, the activities of key enzymes involved in the stress defense systems (*SOD* and *CAT*) were determined at 72 h. In contrast to the H_2_O_2_ content and ROS levels, overall strong *SOD* and *CAT* activity was observed in the elastin peptide-supplemented fermentation cultures. For instance, *SOD* and *CAT* activity reached 673.5 and 308.2 U/g protein at 72 h, which was 68.0 and 70.0% higher than the *SOD* and *CAT* activity of the nonsupplemented group, respectively (Fig. 6d, e). These data were all consistent with the above transcriptome results.

**Fig. 7:**
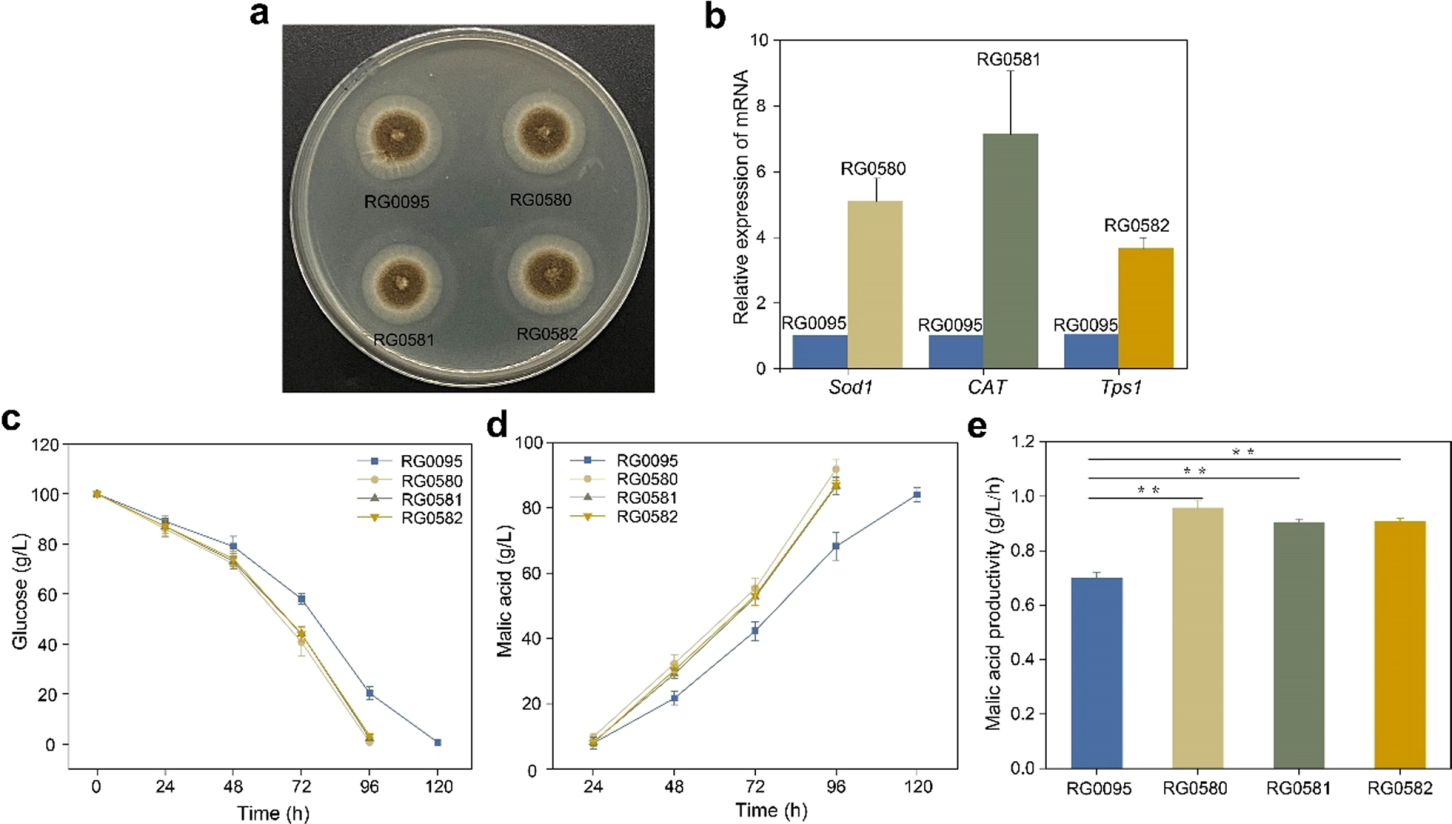
Time course of fermentation of *A. niger* strains. **a** Growth profiles of RG0095, RG0580, RG0581 and RG0582 on potato dextrose agar (PDA) plates at 28 °C for 48 h; **b** expression analysis of the indicated genes by qRT-PCR in RG0095 and RG0580, RG0581 and RG0582; **c** glucose consumption; **d** malic acid production. **e** Malic acid productivity. Data represent the mean ± SD of three independent replicates. Statistical significance was determined by Student’s *t*-test (*n* = 3). *p < 0.05, **p < 0.01

To further investigate the effect of elastin peptide on the enzymes that promote the synthesis of nonenzymatic antioxidants, the *GST* and *GR* activity of *A. niger* treated with the elastin peptide were detected after 72 h of incubation. The *GST* and *GR* activity of the elastin peptide-supplemented cells were higher than the *GST* and *GR* activity of the nonsupplemented cells, with increases of 39.4 and 60%, respectively (Fig. 6f, g). The *GST* activity was again consistent with the transcriptome data. Enzymes such as glutathione S-transferases (*GST*) and glutathione reductase (*GR*) can transform glutathione (GSH) to thiol compounds to scavenge ROS against oxidative stress [33]. In this study, the GSH concentration of elastin peptide supplemented in *A. niger* decreased to 1190.7 µg/g protein at 72 h, which was reduced by 37.6% compared to the nonsupplemented group (Fig. 6h). To this end, we added 2 g/L glutathione (GSH) to 50 mL of fermentation medium, and found that the glucose consumption was slightly higher than that in the nonsupplemented groups after 24 h (Additional file 1: Figure S3a). The GSH supplemented group consumed almost all glucose at 108 h (Additional file 1: Figure S3b), but the strain overexpressing sod1 had residual glucose concentrations of approximately 0.7 g/L at 96 h of fermentation (Fig. 7c). In addition, the GSH added group produced approximately 85.55 g/L malic acid at 108 h and strain overexpressing sod1 exhibited a similar titer of malic acid (91.85 ± 2.58 g/L) (Additional file 1: Figure S3c). However, the productivity of malic acid in GSH supplemented groups (~ 0.79 g/L/h) were higher than the nonsupplemented groups (0.69 ± 0.03 g/L/h). Additionally, the elastin peptide-supplemented group also showed a decline in GSSG content, which was decreased by 59.6% compared with the nonsupplemented group (Fig. 6i). GR converts oxidized glutathione (GSSG) to reduced GSH, thus contributing to endow cells with stress tolerance under stress [34]. Therefore, we further inferred that the consumed GSH and GSSG might combine with intracellular oxidants to reduce stress. Trehalose synthase (*TS*) converts maltose into trehalose by intramolecular transformation [35]. Therefore, we first detected the enzyme (*TS*) that catalyzes trehalose synthesis and found that the TS activity reached 488.6 U/mg protein in the elastin peptide-supplemented cells at 72 h, representing an increase of 82.4% over the nonsupplemented cells (Fig. 6j). Nwaka demonstrated that cytosolic *THL* was responsible for trehalose hydrolysis in *Saccharomyces cerevisiae* cells [36]. Therefore, a decline in trehalase (*THL*) activity was observed in the fermentation medium with elastin peptide at 72 h, and the *THL* activity decreased 245.2 U/mg protein, which was 35.3% lower than that of the nonsupplemented group (Fig. 6k).

To further explore the mechanism of the observed effects of elastin peptide on malic acid production, we determined the key enzymes in the malate synthesis pathway of *A. niger*. For instance, in the glycolysis pathway, after adding elastin peptide, the activities of *HK*, *Pfk* and *PK* were increased by 25.9, 25.8, and 54.2%, respectively (Fig. 6l, n). In addition, we also detected the key enzyme activities (*Pyc* and *MS*) in the r-TCA and glyoxylic acid bypass pathways. As shown in Fig. 6o, p, the pyc and MS activities in the group grown with elastin peptide were increased 52.8 and 54.3% at 72 h of cultivation compared to the nonsupplemented group. These data are generally consistent with the results of transcriptome analysis. In general, the key enzyme activities in glycolysis, r-TCA and the glyoxylic acid bypass pathway of *A. niger* were significantly enhanced and increased the fluxes of the corresponding pathway; thus, the productivity of malic acid in *A. niger* was improved by elastin peptide.

The above results fully proved that the stress resistance of *A. niger* was improved by elastin peptide, such as upregulation of antioxidant enzymes, increase in nonenzymatic antioxidant biosynthesis and downregulation of genes involved in the hydrolysis of antioxidants. Therefore, the oxidative stress of *A. niger* was relieved. At the same time, the metabolism of glucose was accelerated, the expression of enzymes related to malate synthesis was improved, and finally, the synthesis of malic acid was enhanced.

Construction of a highly efficient malic acid productivity in *A. niger*

The above analysis demonstrates that the addition of elastin peptide can alleviate the oxidative stress of *A. niger* during fermentation by directly or indirectly activating antioxidant enzymes. Based on the transcriptome and biochemical analysis data, we found that some stress resistance-related enzymes (e.g., *Sod1*, *Sod2*, *CAT*, *GST* and *Tps1, *etc*.*) were significantly upregulated in the elastin peptide-supplemented group to enhance the stress resistance of cells. Therefore, we can directly overexpress related antioxidant enzymes to improve the resistance of cells. Three antioxidant enzymes (*Sod1*, *CAT*, and *Tps1*) analyzed by the above transcriptome were individually overexpressed in *A. niger* RG0095. The growth of RG0580, RG0581 and RG0582 were identical to that of RG0095 (Fig. 7a). qRT-PCR analysis was used to analyze the resultant transformants to confirm the corresponding overexpressed genes, and the results demonstrated that the transcription levels of these four genes in RG0580, RG0581 and RG0582 were increased by 3.9–6.6 fold compared with the transcription levels in RG0095 (Fig. 7b).

Subsequently, we utilized the transformants for the shake flask fermentation assay, and *A. niger* RG0095 was used as the nonsupplemented group. RG0580, RG0581 and RG0582 had residual glucose concentrations of approximately 0.7, 2.3, and 3.3 g/L, respectively, at 96 h of fermentation (Fig. 7c). In contrast, RG0095 had a residual glucose concentration of 20.3 ± 2.5 g/L at 96 h, and glucose was almost consumed at 120 h (Fig. 7c). Our data showed that the overexpression of *Sod1*, CAT and *Tps1* could shorten the fermentation duration from 120 to 96 h (Fig. 7c). Fortunately, the titers of malic acid from the three overexpression transformants of *Sod1*, CAT and Tps1 maintained the same output as before in 96 h (Fig. 7d). Specifically, the titers of malic acid in cells overexpressing *Sod1*, *CAT* and *Tps1* were 91.85 ± 2.58, 86.65 ± 0.73, and 86.98 ± 1.11 g/L at 96 h, respectively, while the titer of malic acid in RG0095 cells was 83.12 ± 2.54 g/L at 120 h (Fig. 7d). Interestingly, in contrast to RG0095, overexpression of *Sod1* significantly elevated the productivity of malic acid by 39.1% (Fig. 7e). In addition, the productivity of malic acid in the *Sod1-*overexpressing strain was improved by 20% compared to the productivity of malic acid in the elastin peptide-supplemented cells. Therefore, overexpression of stress resistance-related enzymes can achieve or even exceed the effect of peptide addition, that is, alleviate stress and improve the production efficiency of malic acid.

## Discussion

Excessive CO_2_ emissions along with depletion of fossil fuel resources have led to the problems of global warming and energy crisis. Microorganisms were utilized to transform renewing biomass into fuels and chemicals in the field of biorefinery, which provides a perspective orientation to address those concerns [27, 37, 38]. Numerous of microbes have been utilized to produce malic acid by metabolic engineering and fermentation optimization [39–41]. However, the multiple stresses (e.g., oxidative stress, product inhibition and nitrogen limitation) exist in the fermentation process, which inhibiting microbial growth and production, thus finally prolonging fermentation time [14, 15, 17, 19]. To decrease or eliminate the stresses of cell, some peptides were added into the fermentation medium [22–24]. These peptides could effectively relieve stress during microbial fermentation, but the underlying mechanisms of the process were not fully understood.

Satisfactorily, addition of elastin peptide resulted in significantly improvement of malic acid production, indicating that exogenous addition strategies have perplexed effects on cellular metabolism. Elastin peptide is composed of low-molecular-mass peptides and free amino acids (such as alanine, lysine, glycine, valine, proline) [17]. We have also analyzed the elastin peptide compositions (Additional file 1: Table S2). The elastin peptide was found to be composed of a large number of amino acids, among which proline, glycine, glutamate and alanine were the most abundant (Additional file 1: Table S2). These data indicate that the addition of an appropriate amount of amino acids might improve the efficiency of organic acid production by filamentous fungi. The glucose consumption was detected by the following supplementation with 2 g/L of proline, glycine and glutamate, respectively. The glucose consumption in groups individually supplemented with the proline, glycine and glutamate was much higher than that in the nonsupplemented group after 24 h (Additional file 1: Figure S4a). Notably, the groups supplemented with amino acids consumed almost all glucose, whereas the control group had 20.3 ± 2.5 g/L glucose at 96 h (Additional file 1: Figure S4a). Thus, we inferred that proline, glycine and glutamate also promoted glucose utilization in *A. niger*. Next, we tested L-malic acid production in these groups and found that a similar titer of L-malic acid in all amino acids-supplemented group (84.55–88.53 g/L) (Additional file 1: Figure S4b). Our results showed that the addition of amino acids had few effects on the final titers of L-malic acid. Notably, the three amino acids-supplemented groups revealed remarkable productivity of L-malic acid (86.8–92.2 g/L/h), which was nearly 33% higher than in the control group (Figure S4c). Previous studies have showed that L-proline as an osmoprotectant could protect yeast cells from damage of oxidative stress, desiccation, or freezing [42–45]. In addition, L-proline increased protein and membrane stability in low-moisture or high-temperature environments and inhibits aggregation during protein refolding [46, 47]. Therefore, we confirmed that amino acids could relieve the effects of oxidative stress. The mitochondrial activity of sake yeast was stabilized by glycine addition and finally influenced the growth and brewing profiles of yeast [48]. Johann et al. showed that amino acid addition improved heterologous protein production by *S. cerevisiae* [49]. There are no reports have proven that elastic peptide can be directly transported into microbial cells. Therefore, we speculated that the elastin peptide could be hydrolyzed into amino acids after addition to fermentation medium. These data indicated that intracellular L-proline and glycine hydrolyzed from elastin peptide might play an important role in mitigating stress by protecting membrane disorder and protein denaturation during long-term malic acid fermentation.

Previous studies have reported that exogenous nitrogen sources were added for improving the biomass of *S. cerevisiae* [50]. Some amino acids from mixtures of nitrogen sources were utilized and improved the growth rates of strains [51, 52]. Albers and their co-workers showed that exogenous amino acids can be incorporated directly into biomass during fermentation and improve the production of proteins [53]. Therefore, we believed that amino acids as nutrients were used for metabolism in *A. niger*, and thereby enhancing the growth of *A. niger*.

Microbial cells produce ROS and H_2_O_2_ under stressed conditions [54]. Microorganisms have a complete set of antioxidant systems, such as some antioxidant enzymes [55]. When intracellular ROS exceed the normal level and result in a dynamic redox imbalance, the intracellular antioxidant system will remove the excess ROS [56]. As the first line of defense of the antioxidant system, the SOD enzyme is responsible for scavenging superoxide anions and plays a fundamental role in protecting cells from oxidative stress [57, 58]. Catalases are heme-containing antioxidant enzymes and are good scavengers of H_2_O_2;_ catalases degrade hydrogen peroxide into water and oxygen [59]. Moreover, glutathione peroxidases (GSH-Px), as antioxidant enzymes, can scavenge ROS and catalyze the reduction of H_2_O_2_ and organic hydroperoxides by reduced glutathione as the substrate [60]. Glutathione-*S*-transferase (*GST*) is an important defense gene that provides resistance against several stresses, and reducing intracellular stress is achieved mainly by *GST* incorporating reduced glutathione into its target cosubstrates (such as exogenous proteins, xenobiotic compounds, etc.) [61]. Trehalose is a nonreducing disaccharide and has received significant attention for its important roles in protecting membranes and proteins as well as adapting to many stresses, such as cold, drought and oxidative stress [62–65]. Two important enzymes involved in trehalose biosynthesis, that is trehalose 6-phosphate synthase (*Tps1*) and trehalose 6-phosphate phosphatase (*Tps2*) [66]. Previous studies showed that both treatment with endogenous overexpression of *Tps1* and exogenous trehalose could relieve stresses in *Pleurotus ostreatus* [67]. Therefore, catalase (*CAT*), copper/zinc superoxide dismutase (*Sod1*) and trehalose-6-phosphate-synthase (*Tps1*) are crucial for protecting fungal cells against intracellular ROS induced by oxidative stress [68–70].

Organisms have developed diverse strategies to resist ROS-mediated oxidative stresses. During growth, *Sod1* was highly expressed in conidia of *Aspergillus fumigatus*, but the Δ*Sod1* mutant showed a growth inhibition at oxidative stress to menadione [68]. A catalase encoding gene *CatT* could protect *Saccharomyces cerevisiae* cells from the strong oxidative stress and a catalase encoding gene, *cpeB* was deleted in *Aspergillus niger* increased sensitivity to oxidative stress [71, 72]. The survival rate of the Δ*Tps1* mutant after oxidative stress was decreased and *Tps1* was confirmed to protection against oxidative stress in yeast cells [69]. Overexpression of genes encoding antioxidation could protect microorganisms from strong oxidative stress, which has been confirmed in many studies. For instance, endogenous overexpression of *Tps1* could relieve stresses in *Pleurotus ostreatus* [67]*. A. niger* accumulated ROS during malic acid fermentation, which might be related to some stresses, including nutrients depletion, high calcium ion, and products accumulation. Collagen peptide provided *Saccharomyces cerevisiae* with robust stress tolerance and activates antioxidant related genes for enhanced bioethanol production [23]. Peptide supplementation during wild-type *R. oryzae* ATCC 20344 fermentation significantly improved fumaric acid production and the expression of genes encoding antioxidant enzymes and those involved in antistress metabolite biosynthesis were upregulated [24]. In this study, we also found that the level of ROS was decreased and the genes encoding antioxidation were significantly upregulated by adding elastin peptide, and thus the fermentation efficiency of *A. niger* was significantly improved. The above RNA-seq and RT-qPCR analysis found that elastin peptide resulted in the up-regulation of genes such as *Sod1*, *CAT*, and *Tps1*, suggesting that elastin peptide might activate the antioxidant pathway of *A. niger* for alleviating the unfavorable harsh fermentation conditions. With the help of genetic engineering, we overexpressed the genes related to enzymes (*CAT*, *Sod1*, *Tps1*), respectively, which could achieve the effect similar to elastin peptide supplementation. Therefore, we speculated that improving the antioxidant capacity of *A. niger* was closely related to enhance fermentation efficiency. Both wild strains and genetically modified strains can achieve similar effects. In addition, the original exogenous addition method was replaced by genetic modification, and no additional raw material cost was needed. Our investigation is an ideal strategy that can facilitate the further development of successful strategies to reduce the fermentation cost for malic acid production.

In summary, our research envisions that enhancing the antioxidant capacity of *A. niger* could improve the efficiency of malic acid. In our future researches, we also hope to further investigate the role of a certain oxidative stress response protein to oxidative stress in *A. niger*, and analyze how it contributes to alleviate oxidative stress.

## Supplementary Information

**Additional file 1: Figure S1.** Fermentation assessment of different concentration peptide supplemented in *A. niger* bioproduction process. a, glucose consumption kinetic; b, L-malic acid titer. Data represent means +/- SD of three independent replicates. **Figure S2. **Change of DEGs (|log2 (Fold Change) | > 1, P-adjust (FDR) < 0.05) related to organelles in elastin peptide-treated group vs peptide-free group. **Figure S3.** Fermentation kinetics of glutathione supplemented and the overexpressing *Sod1* in *A. niger* RG0095. a, glucose consumption; b, malic acid production; c, malic acid productivity. Data represent the mean ± standard deviation (SD) of three independent replicates. Statistical significance was determined by Student’s t-test (n= 3). **Figure S4.** Fermentation kinetics of different amino acids supplemented in *A. niger* RG0095 fermentation culture medium. a, glucose consumption; b, malic acid production; c, malic acid productivity. Data represent the mean ± standard deviation (SD) of three independent replicates. Statistical significance was determined by Student’s t-test (n= 3). **Table S2.** Types and concentrations of amino acids in elastin peptide.

**Additional file 2: Table S1.** Upregulated and Downregulated DEGs.

## Data Availability

All data generated or analyzed during this study are included in this published article and its supplementary information files.
