# Supplementary material for: Improving the productivity of malic acid by alleviating oxidative stress during Aspergillus niger fermentation
Source: Biotechnol Biofuels Bioprod. 2022 Dec 29;15:151. doi: 10.1186/s13068-022-02250-7 (PMC9801644; doi:10.1186/s13068-022-02250-7)
Supplement: Supplementary file 1 — Additional file 1: Figure S1. Fermentation assessment of different concentration peptide supplemented in A. niger bioproduction process. a, glucose consumption kinetic; b, L-malic acid titer. Data represent means +/- SD of three independent replicates. Figure S2. Change of DEGs (|log2 (Fold Change) | > 1, P-adjust (FDR) < 0.05) related to organelles in elastin peptide-treated group vs peptide-free group. Figure S3. Fermentation kinetics of glutathione supplemented and the overexpressing Sod1 in A. niger RG0095. a, glucose consumption; b, malic acid production; c, malic acid productivity. Data represent the mean ± standard deviation (SD) of three independent replicates. Statistical significance was determined by Student’s t-test (n= 3). Figure S4. Fermentation kinetics of different amino acids supplemented in A. niger RG0095 fermentation culture medium. a, glucose consumption; b, malic acid production; c, malic acid productivity. Data represent the mean ± standard deviation (SD) of three independent replicates. Statistical significance was determined by Student’s t-test (n= 3). Table S2. Types and concentrations of amino acids in elastin peptide. [file 13068_2022_2250_MOESM1_ESM.docx]

**Supplementary material for**

**Improving the productivity of malic acid by alleviating oxidative stress during *Aspergillus niger* fermentation**

Na Wu^1,2^, Mingyan Xing^1^, Yaru Chen^1^, Chi Zhang^1^, Ping Song^1^, Qing Xu^*1^, Hao Liu^3^, He Huang^*1,4^

1 School of Food Science and Pharmaceutical Engineering, Nanjing Normal University, Nanjing 210023, China

2 College of Life Sciences, Nanjing Normal University, Nanjing 210046, China

3 Tianjin Engineering Research Center of Microbial Metabolism and Fermentation Process Control, Tianjin University of Science & Technology, Tianjin 300457, China

4 College of Biotechnology and Pharmaceutical Engineering, Nanjing Tech University, Nanjing 211800, China

*Corresponding author

E-mail: xu_qing@njnu.edu.cn and huangh@njnu.edu.cn


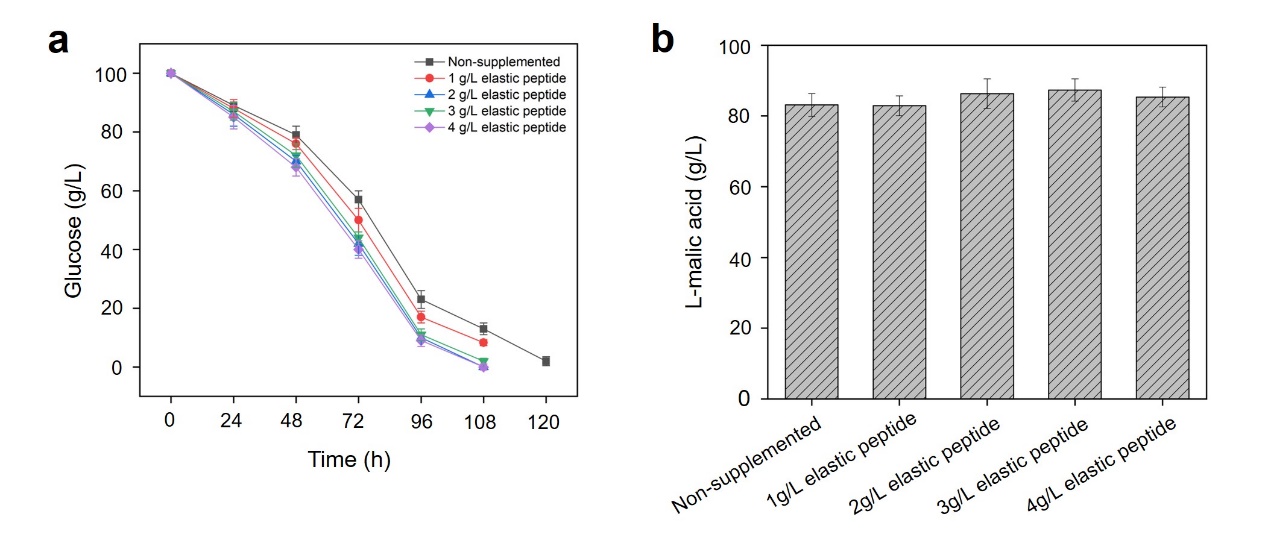


**Figure S1.** Fermentation assessment of different concentration peptide supplemented in *A. niger* bioproduction process. a, glucose consumption kinetic; b, L-malic acid titer. Data represent means +/- SD of three independent replicates.


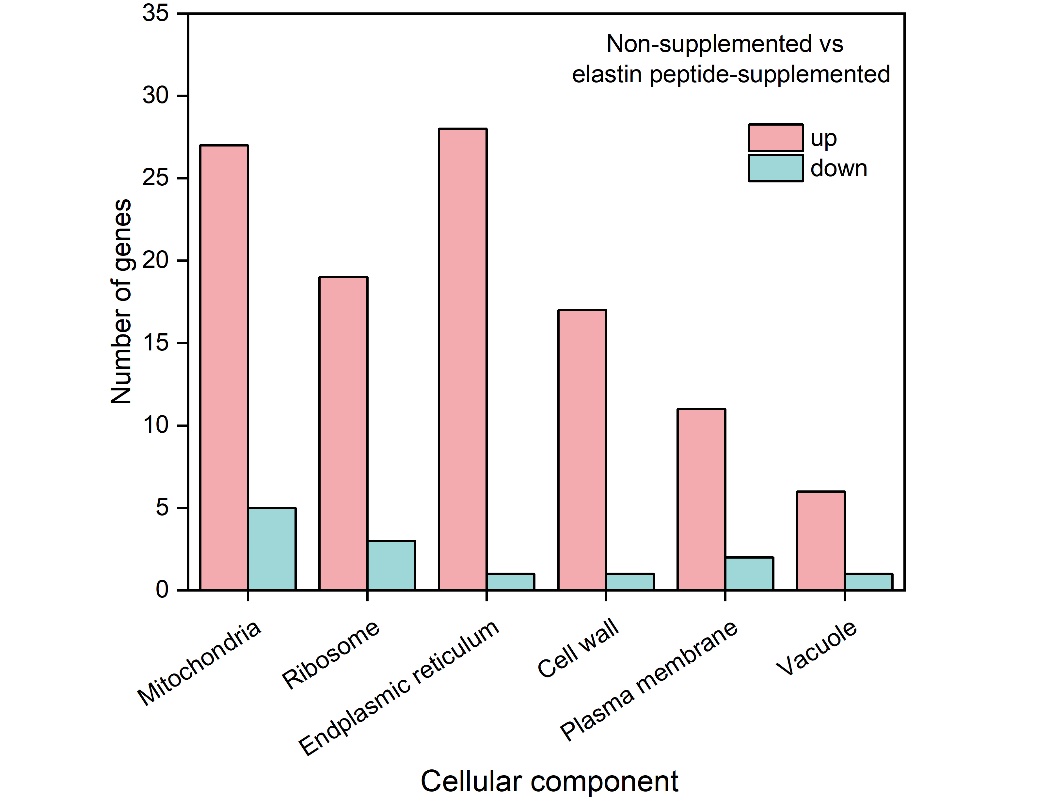


**Figure S2.** Change of DEGs (|log2 (Fold Change) | > 1, P-adjust (FDR) < 0.05) related to organelles in elastin peptide-treated group vs peptide-free group.


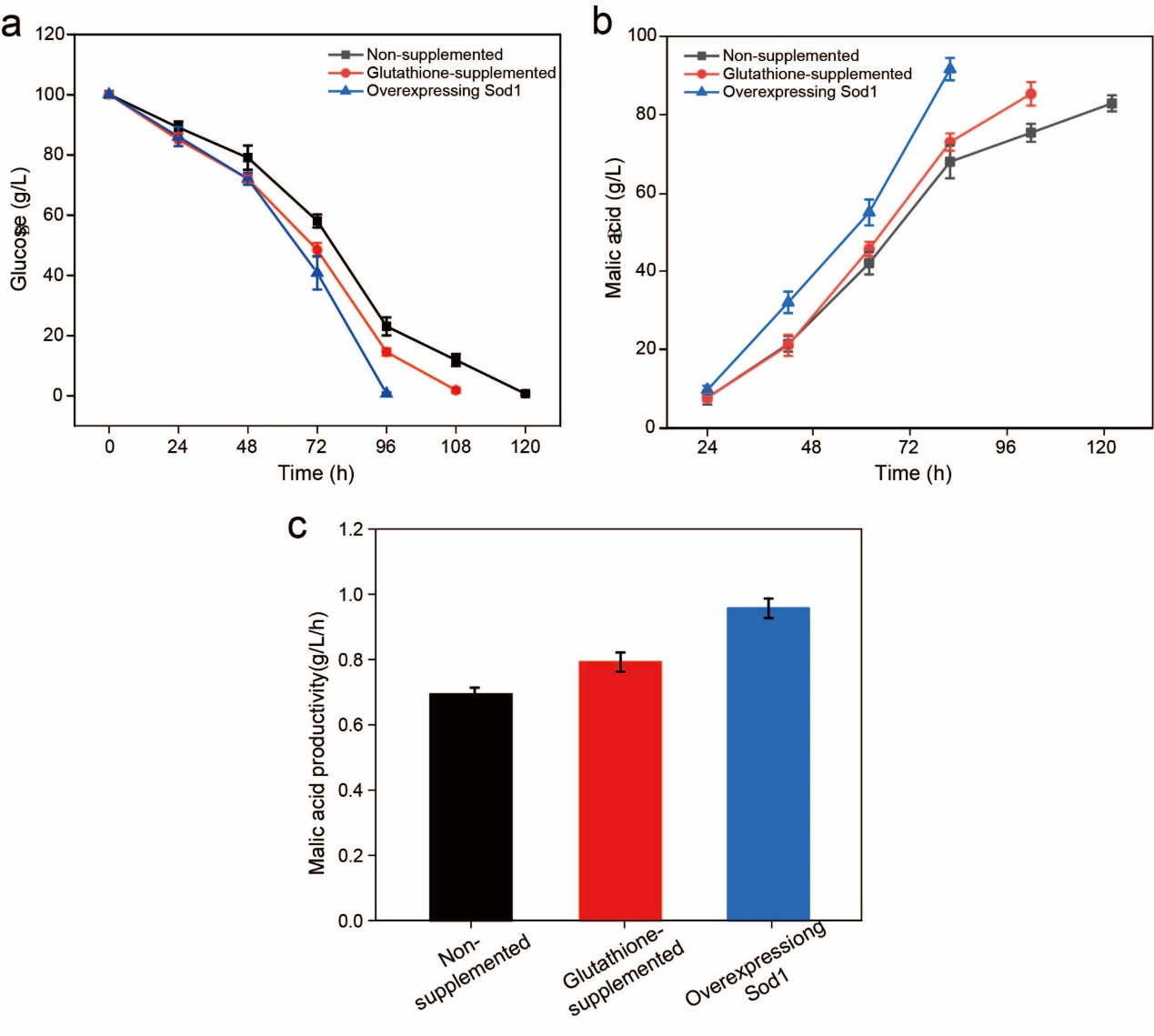


Figure S3. Fermentation kinetics of glutathione supplemented and the overexpressing *Sod1* in *A. niger* RG0095. a, glucose consumption; b, malic acid production; c, malic acid productivity. Data represent the mean ± standard deviation (SD) of three independent replicates. Statistical significance was determined by Student’s t-test (n= 3).

**
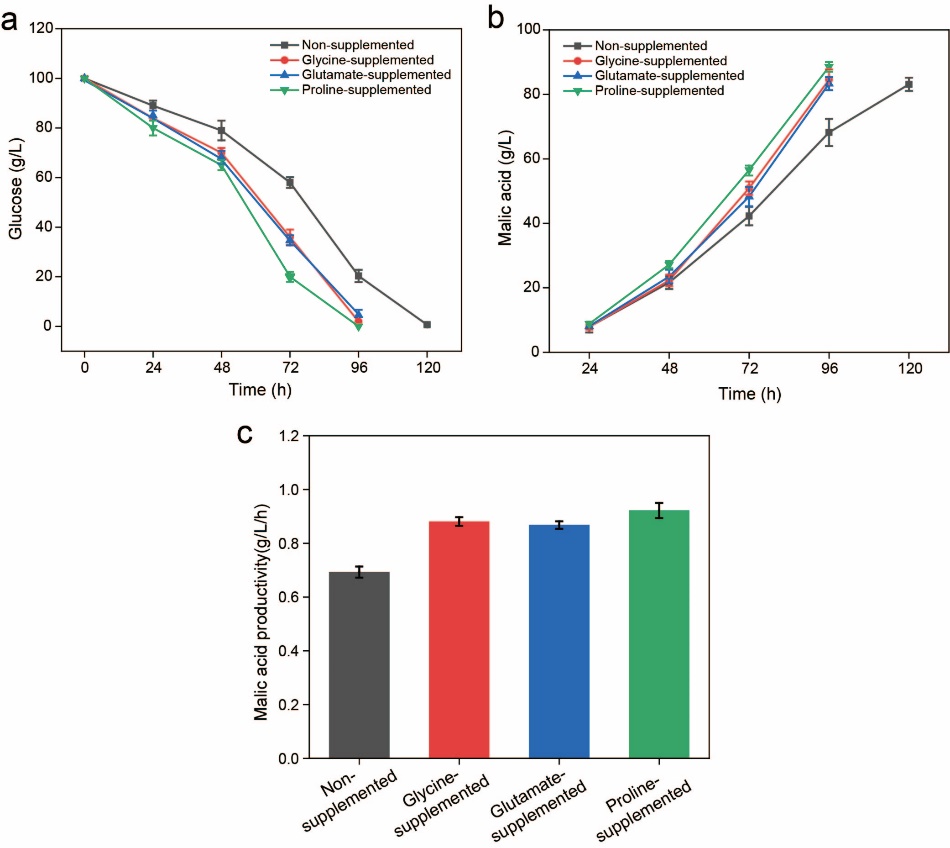
**

Figure S4. Fermentation kinetics of different amino acids supplemented in *A. niger* RG0095 fermentation culture medium. a, glucose consumption; b, malic acid production; c, malic acid productivity. Data represent the mean ± standard deviation (SD) of three independent replicates. Statistical significance was determined by Student’s t-test (n= 3).

Table S3. Types and concentrations of amino acids in elastin peptide.

| Amino acid type | Amino acid concentration (mg/g) |
| --- | --- |
|  | Elastin peptide |
| Pro | 132.7 |
| Gly | 104.1 |
| Glu | 67.6 |
| Ala | 58.1 |
| Arg | 46.8 |
| Asn | 43.5 |
| Met | 40.1 |
| Val | 36.0 |
| Ile | 32.9 |
| Leu | 29.2 |
| Lys | 27.2 |
| Thr | 22.6 |
| Ser | 21.6 |
| Phe | 13.1 |
| His | 9.2 |
| Tyr | 8.1 |
| Cys | 6.6 |
| Total concentration (mg/g) | 699.4 |
